# Latitude and Community Diversity Primarily Explain Invasion Patterns of Widespread Invasive Plants in Small, Subtropical Lakes

**DOI:** 10.1002/ece3.71115

**Published:** 2025-03-13

**Authors:** Samuel A. Schmid, Adrián Lázaro‐Lobo, Cory M. Shoemaker, Andrew Sample, MacKenzie Cade, Gary N. Ervin, Gray Turnage

**Affiliations:** ^1^ Department of Biological Sciences Mississippi State University Mississippi State Mississippi USA; ^2^ Geosystems Research Institute Mississippi State University Starkville Mississippi USA; ^3^ Biodiversity Research Institute (IMIB) University of Oviedo‐CSIC‐Principality of Asturias Mieres Asturias Spain; ^4^ Department of Biology Slippery Rock University Slippery Rock Pennsylvania USA; ^5^ Stantec Consulting Services Inc. Nashville Tennessee USA; ^6^ Truck Crops Branch Experiment Station Mississippi State University Crystal Springs Mississippi USA

**Keywords:** emergent plants, generalized linear models (GLMs), invasive macrophyte, lake perimeter, richness, Secchi depth, species diversity

## Abstract

Within the study of aquatic invasive species, small aquatic ecosystems are often neglected, despite representing most global freshwater bodies. This study uses community composition and environmental and geographic factors to explain the occurrence of invasive species in small lakes in the southeastern United States. Four invasive species widespread in the southeastern United States were selected as the focus of this study: *Alternanthera philoxeroides* , *Cyperus blepharoleptos*, *Panicum repens* , and *Triadica sebifera* . The aquatic plant communities of the lakes were surveyed using littoral zone point sampling. Generalized linear models for each species were fit with the probability of occurrence (*P*_*occ*_) as the response variable and Secchi depth, plant species diversity (*α‐*diversity), point richness, perimeter, latitude, and longitude as potential predictors; all predictors were subjected to model selection to define the best‐fit models. All best‐fit models were strongly predictive with area under the receiver operating characteristic curve values > 0.80. Plant species diversity was positively correlated with *P*_*occ*_ of *A. philoxeroides* , *P. repens* , and *T. sebifera* . Latitude was negatively correlated with *P*_*occ*_ of *P. repens* and *T. sebifera.* Perimeter was negatively related to *P*_*occ*_ of *A. philoxeroides* . Secchi depth was negatively related to the *P*_*occ*_ of *C. blepharoleptos*. Although plant species diversity and latitude were most commonly predictive, *P*_*occ*_ was usually explained by multiple predictors, suggesting that these relationships are best explained with multiple environmental factors.

## Graphical abstract

Invasion patterns of four aquatic plants were predicted using biotic and abiotic factors. Diversity (positively related) and latitude (negatively related) were most commonly predictive of the probability of invasive species occurrence. Invasion patterns for each species were explained by multiple predictors.

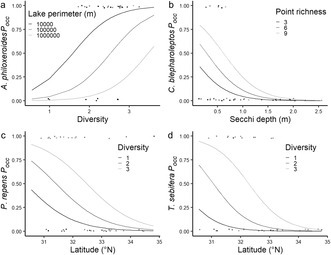

## 1 Introduction

Within the subtropics, there are some of the most culturally and ecologically important freshwater ecosystems in the world (e.g., Mississippi River Alluvial Valley, Nile River, Three Gorges Reservoir; Miranda et al. 2021; Woodward et al. 2022; Liao et al. 2023). However, regardless of importance, one of the greatest threats to subtropical lakes and rivers is invasive species, particularly invasive plants. Subtropical freshwater ecosystems are threatened by several highly invasive plants ( *Alternanthera philoxeroides* , *Pontederia crassipes*, *Salvinia molesta* , etc.) and studying the ecology of these invasive species is critical for mitigating harm to these important freshwater resources (Nega et al. 2022; Hong‐Qun et al. 2023; Holt et al. 2023; Schmid et al. 2025).

Invasive species are costly both monetarily and ecologically, as they alter and often degrade the natural structure and function of the ecosystems they inhabit (Fleming and Dibble 2015; Gallardo et al. 2016; Lázaro‐Lobo and Ervin 2021; Crystal‐Ornelas et al. 2021; Macêdo et al. 2024). Consequently, the field of invasion ecology remains active in research, and research needs are constantly identified and investigated (Kueffer et al. 2013; Fleming and Dibble 2015; Gioria et al. 2023). Within the study of invasion ecology, several hypotheses have been postulated, assessed, and supported through empirical study, which has revealed an intricate complex of factors that describe invasion (Catford et al. 2009; Kueffer et al. 2013; Lowry et al. 2013; Fleming and Dibble 2015; Daly et al. 2023; Gioria et al. 2023). As a result of the complex nature of biological invasion, researchers have outlined the need to investigate multiple invasion hypotheses simultaneously to more completely explain patterns of invasion (Gioria et al. 2023). Additionally, despite the importance of freshwater resources and the deleterious effects of aquatic invasive species, aquatic and wetland ecosystems remain under‐represented in the invasion ecology primary literature (Lowry et al. 2013; Fleming and Dibble 2015; Gallardo et al. 2016; Fleming et al. 2021; Boltovskoy et al. 2021). This study assesses multiple invasion hypotheses, namely how lake ecosystem diversity, geography, and size affect the probability of invasive species occurrence in lakes.

An important, but often overlooked facet of freshwater resources is small, shallow lakes, and these lakes contain the majority of freshwater area on the planet (Scheffer 2004; Downing et al. 2006; Schmid et al. 2022). These ecosystems are often eutrophic and productive with dense aquatic plant communities that provide numerous ecosystem services (Scheffer 2004; Fleming et al. 2021; Ervin 2023). These aquatic plant communities are threatened by invasive plants that tend to displace native species within the ecosystem (Fleming and Dibble 2015; Lázaro‐Lobo and Ervin 2021). While invasive species are a substantial threat to aquatic ecosystems, field studies on invasive plants disproportionately represent terrestrial systems over aquatic and wetland systems (Lowry et al. 2013; Boltovskoy et al. 2021). In addition to the bias toward terrestrial ecosystems, aquatic plant community studies are often focused on large water bodies (Hall and Mills 2000; Santos et al. 2011; Yuan et al. 2013; Cox et al. 2014; Madsen et al. 2021; Philippov et al. 2022). Comparatively, small lakes receive less research attention despite containing more aquatic plant habitat than their larger counterparts (Verhoeven et al. 2020; Lindholm et al. 2021; Schmid et al. 2022). There is a need to improve the scientific understanding of invasive plant ecology within small aquatic ecosystems.

**FIGURE 1:**
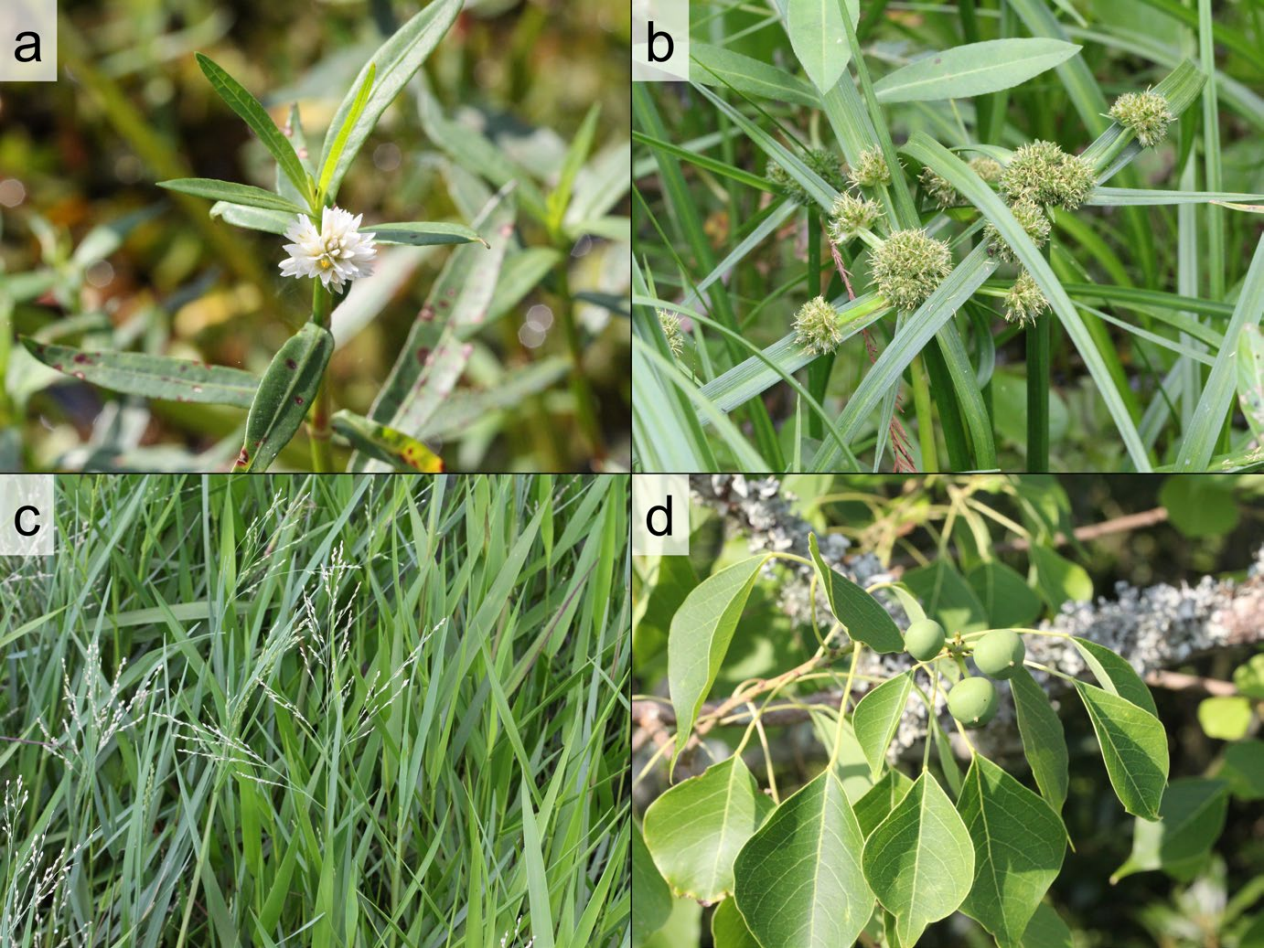
Photos of (a) *Alternanthera philoxeroides* , (b) *Cyperus blepharoleptos*, (c) *Panicum repens* , and (d) *Triadica sebifera* . © Samuel A. Schmid.

The purpose of this study was to assess the applicability of invasion ecology hypotheses on small wetland ecosystems as compared to terrestrial and larger aquatic systems. For this study, we assessed three hypotheses. The first hypothesis states that more species rich and diverse lakes will have a greater probability of occurrence of invasive species. This hypothesis is based on the “rich get richer” concept that states ecosystems with high native richness also tend to support more invasive species (Stohlgren et al. 2003; Fridley et al. 2007; Trotta et al. 2023). The second hypothesis states that geography, specifically latitude, will affect probability of occurrence for invasive species. This is based on two concepts, that invasive species tend to gradually diffuse from their point of introduction and that temperature is a limiting factor for the spread of invasive species (O'Malley et al. 2009; Giometto et al. 2013; Kelley 2014); both of these concepts support a negative relationship between latitude and invasive species probability of occurrence. The third hypothesis states that larger lakes will have greater occurrence of invasive species. The foundation for this hypothesis is the species–area concept which shows environmentally heterogeneous, allowing, conceptually, more opportunities for species coexistence (Gleason 1925; Davies et al. 2005; Catano et al. 2021). This study focuses on four invasive plant species, *A. philoxeroides* (Mart.) Griseb., *Cyperus blepharoleptos* Steud., *Panicum repens* L., and *Triadica sebifera* (L.) Small, all of which are widespread in wetlands throughout the southeastern subtropics of North America (Figure 1).

## 2 Methods

### 2.1 Lake Surveys

**FIGURE 2:**
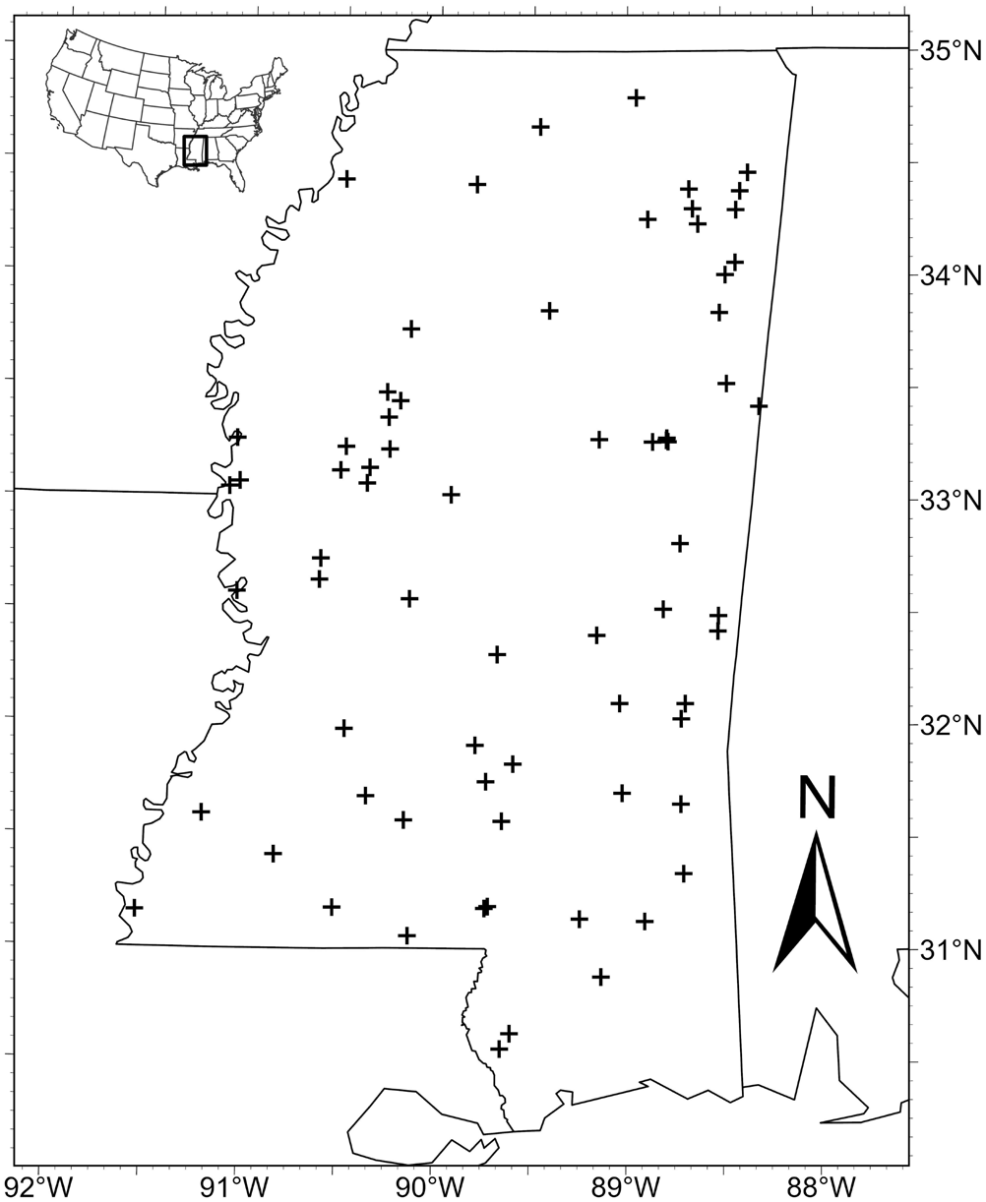
Map of all lakes (*n* = 70) surveyed during June and July of 2017, 2019, 2020, 2022, 2023, and 2024.

During June and July of 2017, 2019, 2020, 2022, 2023, and 2024, 70 lakes in Mississippi, USA, were surveyed (Figure 2). Lake selection focused primarily on small and medium lakes (≤ 1000 ha), specifically to exclude the largest and most‐studied lakes in Mississippi (i.e., Ross Barnett Reservoir, Pickwick Lake, Arkabutla Lake, Grenada Lake, Sardis Lake, and Enid Lake). Aquatic plant communities of selected lakes were surveyed using shoreline point surveys conducted by boat. Similar surveys on larger lakes use the point‐intercept or transect methods to measure within‐lake patterns of plant community composition (Cox et al. 2014; Madsen et al. 2021; Perleberg and Radomski 2021). By comparison, shoreline point surveys allow for sufficient summarization of the littoral zone while reducing survey effort per lake. Survey points were sampled at or near the lake shoreline and were equally spaced at distances between 100 and 1000 m depending on the total length of shoreline (Figure SI1). To survey plant communities, survey points were navigated to, GPS location was logged, and species were recorded. All aquatic plants and charophytes within 3 m of the watercraft were identified in situ and recorded as present (1), and all other species were assumed absent (0) and recorded as such. Plants were identified following Weakley and Southeastern Flora Team (2024), Stotler and Crandall‐Stotler (2017), and Wehr et al. (2015). Emergent and floating species were identified from the watercraft while a plant rake was deployed to sample submersed plants. In addition to surveying the plant community, Secchi depth (m), a measure of water column transparency, was recorded in open water for all lakes, ideally near midday in full sun. All data collection, navigation, and mapping were conducted using ArcGIS Field Maps (Esri 2024) and ArcGIS Pro (Esri 2023).

### 2.2 Ecosystem Data

In addition to recording species presence/absence at survey points, the following plant community indices were calculated for each lake or lake by species combination: species frequency of occurrence (*F*_*i*_), point richness (${\bar{x}}_{s}$), species proportion (*p*_*i*_), and diversity (*H′*). These indices were calculated using the following formulas:

(1) $$F_{i}=\frac{n_{i}}{t}$$

(2) $${\bar{x}}_{s}=\frac{N}{t}$$

(3) $$p_{i}=\frac{n_{i}}{N}$$

(4) $$H^{\prime }=-\sum_{i=1}^{s}\;p_{i}\;\ln\;p_{i}$$

Definition of symbols:

*n*_*i*_ = number of occurrences for species *i*.

*N* = number of occurrences for all species.

*t* = number of survey points.

*t*_*n*_ = number of vegetated survey points.

*s* = species richness.

The value for *H′* was used to represent *α‐*diversity for each lake and was calculated from a modified form of the Shannon–Weiner index that was adapted to the data design of this study (Gotelli and Ellison 2018). For the purposes of this study, *α‐*diversity was used to represent the plant community for the entire lake, whereas point richness was used to represent sub‐community assemblages. Other ecosystem variables were assigned to lakes for this study. These variables include Secchi depth, lake perimeter, latitude, and longitude. Lake perimeter was positively skewed (Figure SI2) and thus log transformed using the natural log function [log_e_(*x*)].

### 2.3 Statistical Analysis

To assess relationships between environmental factors and the invasion patterns of *A. philoxeroides* , *C. blepharoleptos*, *P. repens* , and *T. sebifera* , statistical models were fit to survey data. The binomial response variables for these models were the presence/absence data for each species, which were used with predictors to calculate the probabilities of occurrence (*P*_*occ*_). In total, six environmental variables were identified as potential predictors for these analyses (Table 1). Secchi depth, *α‐*diversity, point richness, log_*e*_(perimeter), latitude, and longitude were all fitted in generalized linear models (GLMs) with logit functions to explain *P*_*occ*_ for *A. philoxeroides* , *C. blepharoleptos*, *P. repens* , and *T. sebifera* . These models were constructed using the “glm()” function in R (R Core Team 2021). Model selection was initiated with an all‐inclusive candidate model consisting of all six predictors. Predictors were removed stepwise using backward selection, where at each step, predictors were removed when the resulting candidate model had the lowest Akaike information criterion (AIC) of all other potential combinations (Dunn and Smyth 2018). The performance of candidate models was principally determined by AIC. In situations where there was substantial support for the consideration of more than one model based on AIC (i.e., ΔAIC ≤ ± 2), model performance was determined based on the *principle of parsimony*, where candidate models with fewer predictors were considered higher performing (Dunn and Smyth 2018). Using these two criteria, the candidate model that performed best was considered our best‐fit model; best‐fit models were determined for *A. philoxeroides* , *C. blepharoleptos*, *P. repens* , and *T. sebifera* . The predictive power of best‐fit models was assessed using the area under the curve (AUC) of the receiver operating characteristic (ROC) curve. Analyses were performed in R (R Core Team 2021) and RStudio (RStudio Team 2020) using base stats functionality and the “pROC” (Robin et al. 2023) packages. For model coefficients, effect significance was determined with *α* = 0.05. Graphing of GLM results was conducted in RStudio using the “ggeffects” (Lüdecke et al. 2023) and “ggplot2” (Wickham et al. 2023) packages.

**TABLE 1:** Names and descriptions of predictors used in this study.

Field	Name	Description
Secchi_depth	Secchi depth (m)	Water clarity as measured with Secchi disk
Diversity	*α*‐Diversity	Adapted Shannon–Weiner species diversity index (*H′*)
Pt_richness	Point richness	Mean number of species at survey points (${\bar{x}}_{s}$)
Log.perimeter	Log_*e*_[perimeter (m)]	Natural log transformation of perimeter of surveyed lakes
Latitude	Latitude (°)	Latitude of site in decimal‐degrees
Longitude	Longitude (°)	Longitude of site in decimal‐degrees

*Note:* Name indicates how variables appear in text and field indicates labels of variables as they appear in figures.

## 3 Results

Statewide, the most common of our four focal species was *A. philoxeroides* , which was recorded at 55 of the 70 lakes, followed by *P. repens* (25 lakes), *T. sebifera* (20 lakes), and *C. blepharoleptos* (13 lakes). These four species were the most common invasive species observed in lake surveys from 2017 to 2024. The mean ($\bar{x}\;\pm \;\mathrm{SD}$) values of model predictors across all lakes surveyed are as follows: Secchi depth (m) = 0.90 ± 0.56, *α‐*diversity = 2.45 ± 0.61, point richness = 4.66 ± 2.30, log_*e*_[perimeter (m)] = 9.45 ± 0.92, latitude (°) = 32.7103 ± 1.1403, and longitude (°) = −89.5606 ± 0.8804.

**FIGURE 3:**
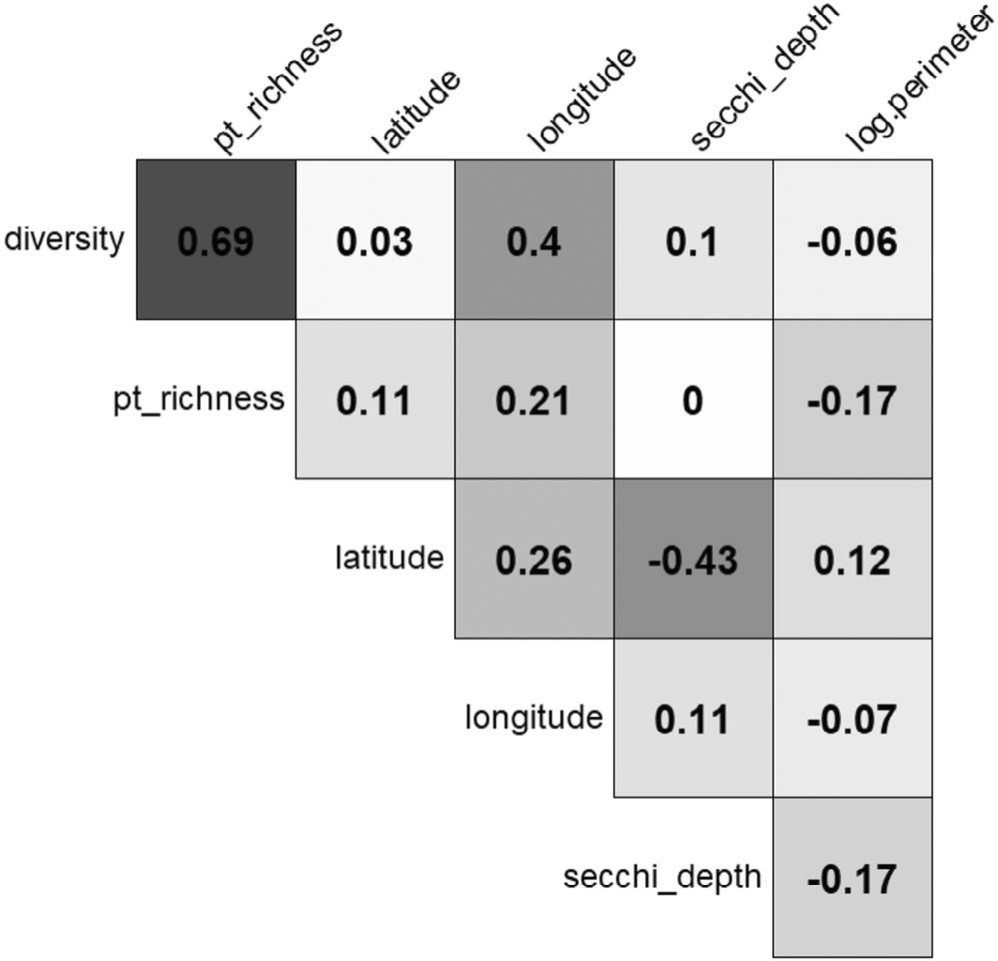
Pairwise Pearson's correlation coefficients (*ρ*) for predictors used in this study (Table 1). Cell darkness corresponds to the *ρ* value. Correlation plot generated in R (R Core Team 2021) using the “corrplot” package (Wei et al. 2021).

**FIGURE 4:**
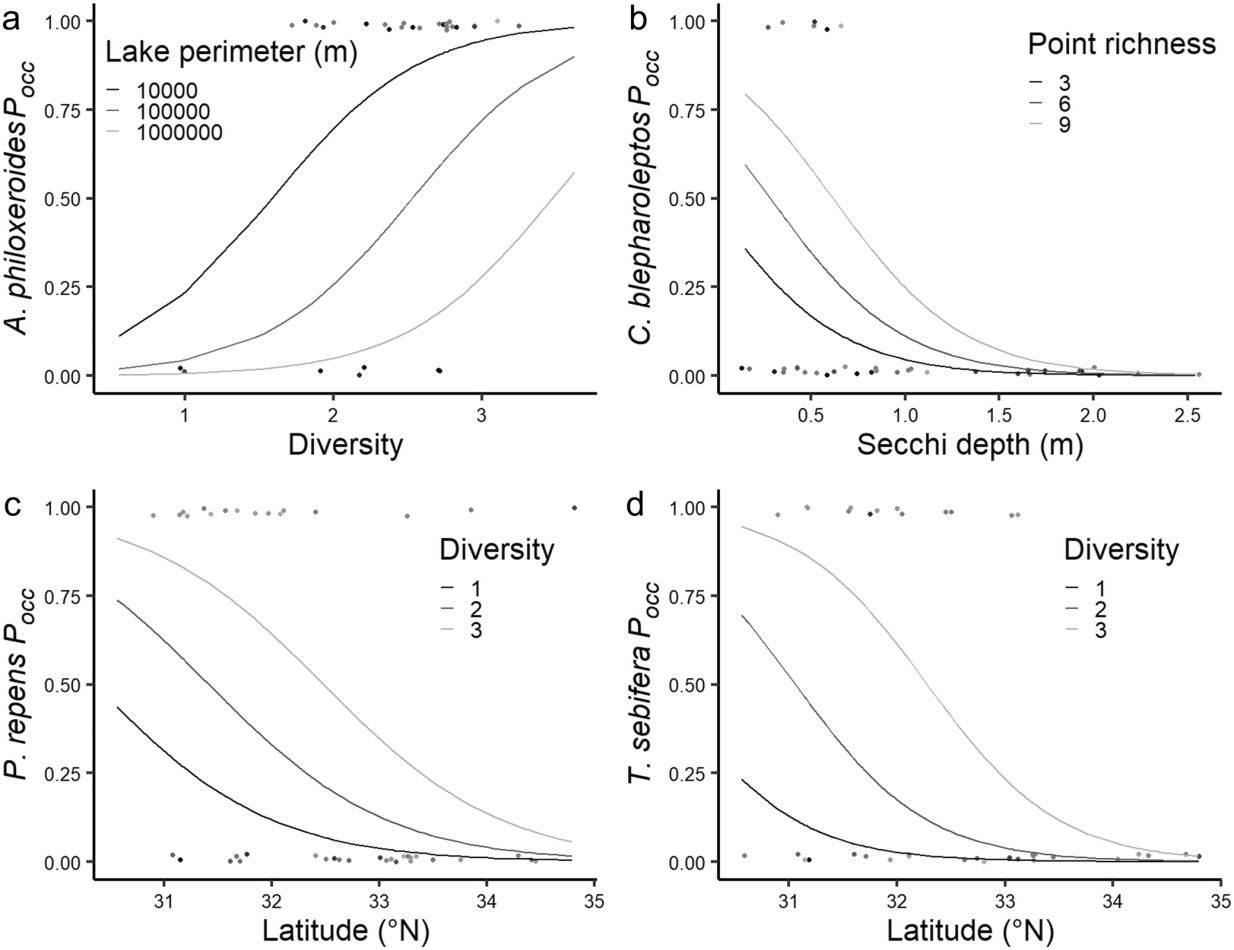
Graphs of best‐fit model functions for how predictor(s) affect the probability of occurrence (*P*_*occ*_) of (a) *A* *lternanthera* *philoxeroides* , (b) *Cyperus blepharoleptos*, (c) *P* *anicum* *repens* , and (d) *T* *riadica* *sebifera* . Graphs show line grayscale groupings that represent the marginal effects of the secondary predictor on the function between the primary predictor and response variable.

Following model selection, the best‐fit model for *A. philoxeroides* consisted of *α‐*diversity and perimeter as predictors (Table SI3). For *C. blepharoleptos*, the best‐fit model consisted of Secchi depth and point richness as predictors (Table SI3). The highest performance models for *P. repens* and *T. sebifera* both consisted of *α‐*diversity and latitude as predictors (Table SI3). To account for spatial autocorrelation, both the *P. repens* and *T. sebifera* highest performance models were compared to models which substituted latitude for a latitude × longitude interaction effect; however, in both instances, the interaction effect was not significant, and the highest performance models were accepted as the best‐fit models. All best‐fit models contained two significant predictors (Table 2). For *A. philoxeroides* , *P*_*occ*_ was positively related to *α‐*diversity and negatively related to perimeter (Table 2; Figure 3a). For both *P. repens* and *T. sebifera* , *P*_*occ*_ was positively related to *α‐*diversity and negatively related to latitude (Table 2; Figure 4c,d). For *P. repens* and *T. sebifera* , the effect size of latitude was greater than *α‐*diversity, whereas for *A. philoxeroides* , *α‐*diversity had the larger effect size (Table 2). For *C. blepharoleptos*, *P*_*occ*_ was negatively related to Secchi depth and positively related to point richness (Table 2; Figure 4b). All best‐fit models were highly predictive over the null model with AUC > 0.80 (Table 2).

**TABLE 2:** Metrics of best‐fit models for *A* *lternanthera* *philoxeroides* , *Cyperus blepharoleptos*, *P* *anicum* *repens* , and *T* *riadica* *sebifera* .

Species	Predictor	Slope coeff	df	*z*‐value	*p*	AUC
*Alternanthera philoxeroides*	Diversity	2.006	69	3.093	0.002	0.825
	Log_*e*_(perimeter)	−0.823	69	−2.171	0.030	
*Cyperus blepharoleptos*	Secchi depth	−2.896	69	−2.342	0.019	0.822
	Point richness	0.322	69	2.163	0.031	
*Panicum repens*	Latitude	−1.216	69	−3.732	< 0.001	0.865
	Diversity	1.296	69	2.220	0.026	
*Triadica sebifera*	Latitude	−1.654	69	−3.791	< 0.001	0.885
	Diversity	2.015	69	2.644	0.008	

*Note:* Shown are the predictors included in best‐fit models along with their respective slope coefficients (slope coeff.), degrees of freedom (df), *z*‐values, and *p* values. Also included is the receiver operating characteristic curve area under the curve (AUC).

## 4 Discussion

Our first hypothesis (i.e., richer systems have greater probability of occurrence of invasive species) was partially supported by the best‐fit models for *A. philoxeroides* , *P. repens* , and *T. sebifera* . Our second hypothesis (i.e., latitude affects probability of occurrence) was partially supported by the best‐fit models for *P. repens* and *T. sebifera* . Our final hypothesis (i.e., larger lakes had greater probability of occurrence) was partially refuted by the best‐fit model for *A. philoxeroides*. Generally, in modeling the invasion patterns of these four species across small Mississippi lakes, we observed some broader trends in the factors that affect *P*_*occ*_ while some effects were species‐specific.

Our first hypothesis was principally based on the “rich get richer” hypothesis; this hypothesis was predicated on a strong, positive‐correlational pattern between native species richness and non‐native species richness across the United States (Lonsdale 1999; Stohlgren et al. 2003; Fleming et al. 2021). Simply, this relationship is due to native‐species–rich ecosystems supporting greater numbers of non‐native species because these systems have greater environmental heterogeneity (Davis et al. 2000; Stohlgren et al. 2003; Lázaro‐Lobo et al. 2020). Our hypothesis supposes a logical next‐step where the probability of occurrence for specific invasive species is greater in more diverse communities. The relationship between native species richness and introduced species richness has endured a falsely dichotomous debate between the “rich get richer” hypothesis and the “diversity barrier” hypothesis. The diversity barrier hypothesis supposes that more diverse ecosystems are more resistant to invasion because there is less niche space available, and this effect is supported by several ecological experiments (Elton 1958; Levine and D'Antonio 1999; Li et al. 2022). However, several authors have discussed that both these patterns are observed simultaneously, and at different spatial scales (Kennedy et al. 2002; Dietz and Edwards 2006; Pauchard and Shea 2006; Fridley et al. 2007; Guo et al. 2023). That is, at smaller, sub‐community spatio‐temporal scales, more diverse species assemblages are more resistant to invasion, but at larger scales, more diverse communities are more likely to be invaded (Kennedy et al. 2002; Dietz and Edwards 2006; Pauchard and Shea 2006). Due to the more regional scope of our study, our findings are more consistent with the “rich get richer” hypothesis (Stohlgren et al. 2003; Trotta et al. 2023). Our first hypothesis was supported by our *A. philoxeroides* , *P. repens* , and *T. sebifera* best‐fit models (Table 2; Figure 4a,c,d). In this study, *C. blepharoleptos P*_*occ*_ was inconsistent with these patterns as it was not predicted by *α‐*diversity and was positively predicted by point richness. When differentiating between native and introduced species, the *C. blepharoleptos* frequencies of occurrence (*F*_*i*_) were more correlated to the point richness of native species (*ρ* = 0.458) than the point richness of introduced species (*ρ* = 0.225). These values suggest that this case was simply an exception to the “Biodiversity Barrier” hypothesis, rather than an instance of invasion meltdown (Simberloff and Von Holle 1999; Kennedy et al. 2002; Fleming and Dibble 2015). These findings warrant more thorough investigation of this phenomenon.

Our second tested hypothesis that latitude affects the invasive probability of occurrence can be explained through two possible mechanisms. The first possible mechanism can be explained by the tropical/subtropical origins of (most of) these invasive species (Bryson et al. 1996, 2008; Bruce et al. 1997; Tanveer et al. 2018); *P. repens* is more widely distributed in Mediterranean Europe, but in the United States it is primarily relegated to the southeastern states, particularly the Gulf Coast states (Enloe and Netherland 2017; Zuloaga et al. 2018; Sperry et al. 2023). Several niche modeling studies have been conducted on these species, and multiple models predict cold temperatures as a limiting factor in these species (Wilcut et al. 1988; Sánchez‐Restrepo et al. 2023; Squires et al. 2024; Liu et al. 2024; Schmid et al. 2025). In this study system, where temperature drives *P*_*occ*_, we would expect to see a negative relationship between latitude and *P*_*occ*_ across the state of Mississippi. The second possible mechanism for hypothesis two was based on the tendency toward diffusion of invasive species across space and time from the point of introduction; this process is an application of the Fisher–Kolmogorov equation to invasion systems (Kolmogorov et al. 1937; Neubert and Parker 2004; Giometto et al. 2013; Huang and Zhang 2021). This application of the Fisher–Kolmogorov equation supposes that an invasive species' population density (*n*; assumed related to *P*_*occ*_) at *x* distance from invasion origin *x*_*0*_ will increase with increasing time (*t*); consequently, *n* at *t* will decrease with increasing *x* (Kolmogorov et al. 1937; Neubert and Parker 2004; Huang and Zhang 2021). Our hypothesis then assumes that increasing distance (in latitude and/or longitude) from initial introduction would negatively affect *P*_*occ*_. While the initial introductions for *P. repens* and *C. blepharoleptos* remain cryptic, both *A. philoxeroides* (Mobile, AL) and *T. sebifera* (Savannah, GA) are believed to have been initially introduced at very southern latitudes (Bruce et al. 1997; Cohen GH01928487; Tanveer et al. 2018). Notwithstanding the uncertainty around introduction, all four of these species are infamously problematic in the southern latitudes around the northern coast of the Gulf of Mexico (Wilcut et al. 1988; Bruce et al. 1997; Bryson et al. 2008; Tanveer et al. 2018). In the context of Mississippi lakes, the primary direction of diffusion for these species is northward; the second hypothesis would consequently predict that *P*_*occ*_ decreases with increasing latitude. Regardless of the active mechanism driving this hypothesis, the negative effect of latitude on *P*_*occ*_ was observed in the best‐fit models for both *P. repens* and *T. sebifera* (Table 2; Figure 4c,d) which support our second hypothesis. Neither the *A. philoxeroides* nor the *C. blepharoleptos* models supported this second hypothesis.

Our third tested hypothesis was premised on a similar mechanism to our first hypothesis that larger lakes will have richer communities and, therefore, a greater probability of occurrence of invasive species (Fleming et al. 2021). This concept is based on the foundational plant ecology axiom that species richness increases with increasing area (*et ergo*, perimeter); and this pattern is driven by increased environmental heterogeneity with increasing spatial area (and perimeter; Gleason 1925; Davies et al. 2005; Fridley et al. 2007; Catano et al. 2021). This increased environmental heterogeneity is predicted to increase the *P*_*occ*_ of these invasive species, as would be expected by the rich get richer hypothesis (Stohlgren et al. 2003; Fleming et al. 2021). From our best‐fit models, only the *A. philoxeroides* model had perimeter as a significant predictor; however, *A. philoxeroides* was more likely to occur in smaller lakes than in larger lakes (although the effect size of diversity was substantially greater than that of perimeter). A possible explanation for why the *A. philoxeroides* model did not conform to our hypothesis is that, for the lakes studied, perimeter was not correlated to *α*‐diversity (*ρ* = −0.06; Figure 3). One potential cause of this relationship is that the lakes included both natural and artificial waterbodies. Another study on Mississippi lakes found that whether a lake was natural or artificial determined the physical and chemical composition (i.e., Secchi depth and nutrients) as well as fish assemblages (Miranda et al. 2018). While that study focused on fishes, the same factors could very feasibly influence the aquatic plant community (Miranda et al. 2018). Alternatively, Mississippi lakes could be an exception to the species‐area relationship that is thoroughly supported in other systems (Gleason 1925; Fridley et al. 2007; Catano et al. 2021). Although the effect of lake perimeter on invasion is not thoroughly studied, lake fetch has demonstrated a positive (albeit nonlinear) effect on the *P*_*occ*_ of invasive species (Fleming et al. 2021). The negative effect of perimeter on *A. philoxeroides P*_*occ*_ is an exception to our current understanding of invasive plant ecology, and further investigation is required to better describe this relationship.

Of the subject species in this study, *C. blepharoleptos* was the only species that neither supported nor refuted any of our tested hypotheses. Instead, in the best‐fit model, the *P*_*occ*_ of *C. blepharoleptos* was negatively related to Secchi depth (and positively related to point richness); that is, *C. blepharoleptos* more commonly occurred in more turbid lakes. Light is well known as a limiting resource for primary producers in aquatic systems, and this relationship is often driven by turbidity (Lacoul and Freedman 2006; Bornette and Puijalon 2011; Schmid et al. 2022). However, for this study, Secchi depth was included as a null predictor as light is not a limiting factor for emergent vegetation, so this result opposes expectations. One possible explanation for this finding is that Secchi depth in this context is unimportant as a measure of turbidity but instead acts as a proxy for a more influential environmental factor. For instance, the correlative relationship among Secchi depth, dissolved organic matter (DOM), and phosphorous is a foundational principle in limnology (Carlson 1977; Nürnberg and Shaw 1998; Wetzel 2001; Golubkov and Golubkov 2024). Within DOM, chlorophyll *a* is a particularly important component as it is a primary photosynthetic pigment in most freshwater algae and cyanobacteria (Nürnberg and Shaw 1998; Wetzel 2001; Golubkov and Golubkov 2024). Secchi depth in this instance could indicate greater nutrient availability, which could be beneficial for the establishment of *C. blepharoleptos*. However, Secchi depth is not a perfect proxy for nutrient availability and may also measure fine‐texture suspended sediments (Lind 1986; Wetzel 2001; Golubkov and Golubkov 2024). In this case, Secchi depth could also indicate the fineness of the benthic sediment, a property that has demonstrated effects on the aquatic plant community structure in previous research (Case and Madsen 2004; Liu et al. 2017; Schmid et al. 2022). As an epiphyte, *C. blepharoleptos* often relies on other aquatic vegetation to establish in an ecosystem, so it presumably depends on aquatic plant community structure, but this relationship requires further investigation (Bryson et al. 2008). Of the species in this study, *C. blepharoleptos* was observed at the fewest lakes, and this species is often considered an emerging invasive species (Bryson et al. 2008; Squires et al. 2024). It is possible that as *C. blepharoleptos* is observed at more lakes in the future, the best‐fit model would identify different significant predictors. Regardless, the relationship between Secchi depth and *C. blepharoleptos P*_*occ*_ is apparent; however, the true nature of this relationship remains unknown.

The results from this study support multiple hypotheses on invasion ecology by examining the regional patterns of *P*_*occ*_ in four specific invasive species. The ecological principals that affect invasiveness in plants have been thoroughly reviewed in the scientific literature and the consensus is that invasion is most often explained through several hypotheses (Catford et al. 2009; Fleming and Dibble 2015; Daly et al. 2023; Gioria et al. 2023). Consequently, there is a considerable research need for empirical studies that assess multiple invasion hypotheses (Catford et al. 2009; Fleming and Dibble 2015; Gioria et al. 2023). Specifically, of invasion hypotheses tested in this study our findings suggest that the “rich get richer” hypothesis best predicted patterns of invasion among species (Stohlgren et al. 2003; Fridley et al. 2007; Trotta et al. 2023). While the results from this study are compelling, the factors that govern invasion patterns are complex and multifaceted, and the relative importance of these different factors remains somewhat obscure. Future research should investigate a wider breadth of factors, including but not limited to: water quality data, sediment characteristics, land use, and vegetation cover. Nevertheless, the effects examined in this study help elucidate which of the processes examined help explain the occurrence of these invasive species in these regional ecosystems. Unfortunately, however, the design of this study provides no information on the degree of invasiveness (i.e., density, abundance, frequency) of these species in these systems. Further investigations should focus on the factors that predict these species' degree of invasiveness. Future research on the patterns of aquatic plant invasion also need to be broadened both taxonomically and geographically. Current research on these patterns is often limited in either taxonomic (Gillard et al. 2017; Yuan et al. 2021) or geographic (Lech and Willig 2021; Wang et al. 2022; Nunes et al. 2022) scope.

These findings help improve our understanding of the factors that influence aquatic plant invasion patterns in Mississippi specifically, and the greater Southeast more broadly as these species are widely distributed in the southeastern United States. This information is valuable for the regional management of aquatic invasive species. Resource managers can use these findings to direct their early detection and prevention efforts for these species. Our results suggest that management efforts focused on *A. philoxeroides* , *P. repens* , and *T. sebifera* should prioritize more diverse communities. Specifically, with *P. repens* and *T. sebifera* , efforts should be focused at more southern latitudes. With *C. blepharoleptos*, the small number of invaded lakes makes it difficult to be prescriptive; however, our findings suggest early detection should prioritize more turbid ecosystems. This application should also remain flexible as the measurable predictors of *C. blepharoleptos* invasion may change as its range expands.

## Author Contributions

**Samuel A. Schmid:** conceptualization (equal), data curation (lead), formal analysis (lead), investigation (lead), methodology (equal), validation (lead), visualization (lead), writing – original draft (lead), writing – review and editing (lead). **Adrián Lázaro‐Lobo:** methodology (equal), writing – review and editing (supporting). **Cory M. Shoemaker:** methodology (equal), writing – review and editing (supporting). **Andrew Sample:** methodology (equal), writing – review and editing (supporting). **MacKenzie Cade:** methodology (equal), writing – review and editing (supporting). **Gary N. Ervin:** conceptualization (equal), writing – original draft (supporting), writing – review and editing (supporting). **Gray Turnage:** conceptualization (equal), funding acquisition (lead), project administration (lead), resources (lead), supervision (lead), writing – original draft (supporting), writing – review and editing (supporting).

## Conflicts of Interest

The authors declare no conflicts of interest.

## Supporting information


Data S1:

## Data Availability

Data and code used in this study are publicly available at the Mississippi State University institutional repository (https://doi.org/10.54718/UEGL1620). All other reasonable requests can be directed to the corresponding author.
