# Supplementary material for: Latitude and Community Diversity Primarily Explain Invasion Patterns of Widespread Invasive Plants in Small, Subtropical Lakes
Source: Ecol Evol. 2025 Mar 13;15(3):e71115. doi: 10.1002/ece3.71115 (PMC11904311; doi:10.1002/ece3.71115)
Supplement: Supplementary file 1 — Data S1: [file ECE3-15-e71115-s001.docx]

**Latitude and community diversity primarily explain invasion patterns of widespread invasive plants in small, subtropical lakes.**

Samuel A. Schmid^1,2*^, Adrián Lázaro-Lobo^3^, Cory Shoemaker^4^, Andrew Sample^5^, MacKenzie Cade^6^, Gray Turnage^2^, and Gary N. Ervin^1^

^*^corresponding author: samuel.schmid@msstate.edu

^1^Mississippi State University, Department of Biological Sciences, Miss State, MS, USA

^2^Mississippi State University, Geosystems Research Institute, Starkville, MS, USA

^3^Universidad de Oviedo, Departamento de Biología de Organismos y Sistemas, Oviedo, Spain ^4^Slippery Rock University, Deptartment of Biology, Slippery Rock, PA, USA

^5^Stantec, Inc., Nashville, TN, USA

^6^Mississippi State University, Mississippi Agriculture and Forestry Experiment Station, Crystal Springs, MS, USA


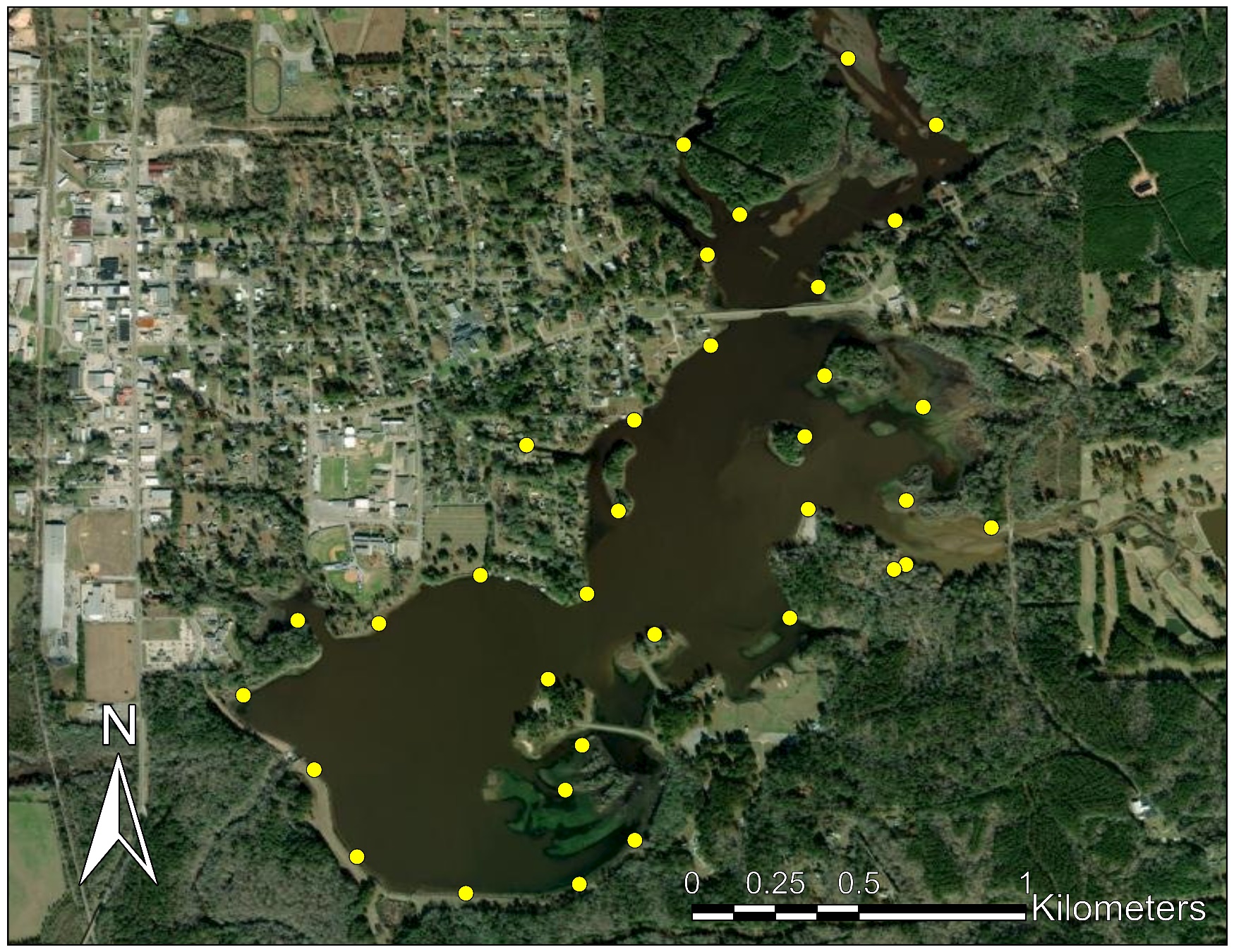


Fig SI1. Example of survey points (n = 34) at Archusa Creek Lake surveyed in June 2023.


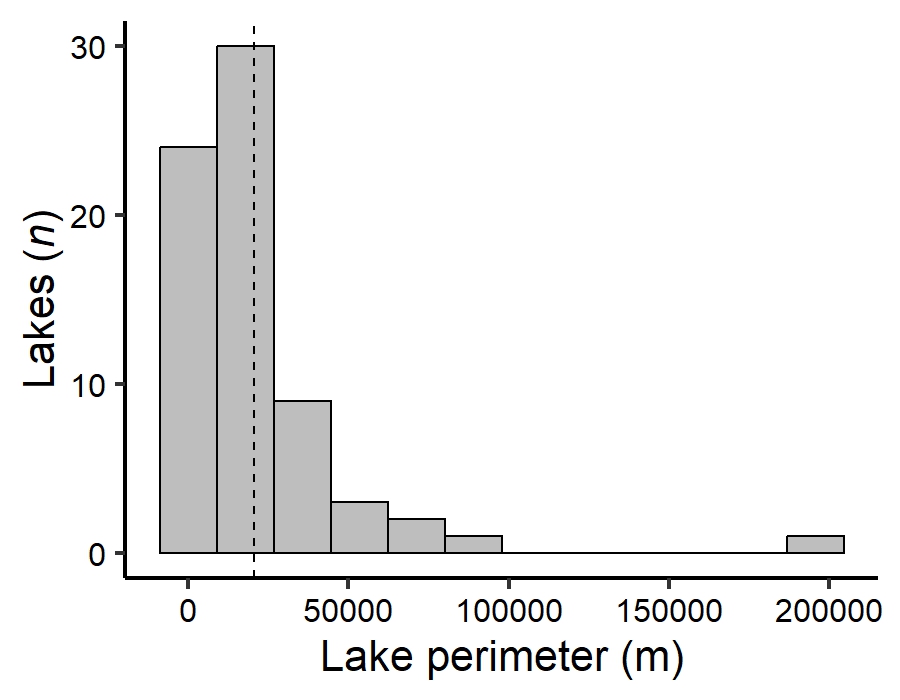
Fig SI2. Histogram of lake perimeter (m) across all lakes surveyed from 2017-2024. Dashed line indicates mean lake perimeter (*x̄* = 20480 m).

Table SI3. Model predictors and Akaike information criterion (AIC) for all candidate models from model selection for *A. philoxeroides, C. blepharoleptos, P. repens,* and *T. sebifera.* Model rank was determined with a combination of AIC and model parsimony. Rank 1 models for each species were selected as the best-fit model.

| Species | Candidate model | AIC | ΔAIC | Rank |
| --- | --- | --- | --- | --- |
| *Alternanthera philoxeroides* | log*_e_*(perimeter) + diversity | 61.654 | 0.000 | 1 |
|  | log*_e_*(perimeter) + latitude + diversity | 60.268 | -1.386 | 2 |
|  | log*_e_*(perimeter) + latitude + longitude + diversity | 61.431 | -0.223 | 3 |
|  | log*_e_*(perimeter) | 64.620 | 2.966 | 4 |
|  | log*_e_*(perimeter) + latitude + longitude + point richness + diversity | 63.268 | 1.614 | 5 |
|  | Secchi depth + log*_e_*(perimeter) + latitude + longitude + point richness + diversity | 65.229 | 3.575 | 6 |
| *Cyperus blepharoleptos* | Secchi depth + point richness | 57.225 | 0.000 | 1 |
|  | Secchi depth + log*_e_*(perimeter) + point richness | 56.228 | -0.997 | 2 |
|  | Secchi depth + log*_e_*(perimeter) + latitude + point richness | 57.078 | -0.147 | 3 |
|  | Secchi depth + log*_e_*(perimeter) + latitude + longitude + point richness | 57.249 | 0.024 | 4 |
|  | Secchi depth + log*_e_*(perimeter) + latitude + longitude + point richness + diversity | 58.377 | 1.152 | 5 |
|  | Secchi depth | 60.498 | 3.273 | 6 |
| *Panicum repens* | latitude + diversity | 73.244 | 0.000 | 1 |
|  | latitude + point richness + diversity | 73.013 | -0.231 | 2 |
|  | latitude + longitude + point richness + diversity | 74.284 | 1.040 | 3 |
|  | latitude | 77.012 | 3.768 | 4 |
|  | log*_e_*(perimeter) + latitude + longitude + point richness + diversity | 75.647 | 2.403 | 5 |
|  | Secchi depth + log*_e_*(perimeter) + latitude + longitude + point richness + diversity | 77.310 | 4.066 | 6 |
| *Triadica sebifera* | latitude + diversity | 58.323 | 0.000 | 1 |
|  | latitude + longitude + diversity | 58.218 | -0.105 | 2 |
|  | latitude + longitude + point richness + diversity | 58.410 | 0.087 | 3 |
|  | log*_e_*(perimeter) + latitude + longitude + point richness + diversity | 60.113 | 1.790 | 4 |
|  | Secchi depth + log*_e_*(perimeter) + latitude + longitude + point richness + diversity | 62.072 | 3.749 | 5 |
|  | latitude | 65.988 | 7.665 | 6 |
